# Food Byproducts as Sustainable Ingredients for Innovative and Healthy Dairy Foods

**DOI:** 10.3390/nu10101358

**Published:** 2018-09-22

**Authors:** Maite Iriondo-DeHond, Eugenio Miguel, María Dolores del Castillo

**Affiliations:** 1 Instituto Madrileño de Investigación y Desarrollo Rural, Agrario y Alimentario (IMIDRA), N-II km 38,200, 28800 Alcalá de Henares, Spain; maite.iriondo@madrid.org (M.I.-D.); eugenio.miguel@madrid.org (E.M.); 2 Instituto de Investigación en Ciencias de la Alimentación (CIAL) (CSIC-UAM), C/ Nicolás Cabrera, 9, Campus de la Universidad Autónoma de Madrid, 28049 Madrid, Spain

**Keywords:** byproducts, sustainability, functional foods, dairy products

## Abstract

The valorization of food wastes and byproducts has become a major subject of research to improve the sustainability of the food chain. This narrative review provides an overview of the current trends in the use of food byproducts in the development of dairy foods. We revised the latest data on food loss generation, the group of byproducts most used as ingredients in dairy product development, and their function within the food matrix. We also address the challenges associated with the sensory properties of the new products including ingredients obtained from byproducts, and consumers’ attitudes towards these sustainable novel dairy foods. Overall, 50 studies supported the tremendous potential of the application of food byproducts (mainly those from plant-origin) in dairy foods as ingredients. There are promising results for their utilization as food additives for technological purposes, and as sources of bioactive compounds to enhance the health-promoting properties of dairy products. However, food technologists, nutritionists and sensory scientists should work together to face the challenge of improving the palatability and consumer acceptance of these novel and sustainable dairy foods.

## 1. Introduction

Sustainability presents both an opportunity and a challenge to the dairy sector. It is an opportunity, because the possibility of using food-processing byproducts for bioactive compound and nutrient extraction has created enormous scope for waste reduction and indirect income generation [1]. However, the challenge is to sustainably intensify the global food production system to enhance food security and nutrition without sacrificing the environment, and to render the concept of sustainable functional foods into a marketable product that is acceptable to consumers [2,3]. 

The development of novel food and/or functional food products is increasingly challenging, as it has to fulfill the consumer’s expectations for products that are simultaneously palatable and healthy [4]. Compared to conventional foods, the development of functional components and technological solutions can be demanding and expensive, and needs of a tight strategy between research and business. All this occurs in a context where functional food markets are continuously changing [5,6]. 

The purpose of this review is to summarize the research findings on the application of various food-processing byproducts used as a source of targeted compounds or as whole ingredients in the manufacturing of dairy foods. So far, most studies available on the valorization of agro-industrial food wastes focus on specific byproducts and their applications in different foods. In this review, the focus is on dairy product development, and how byproducts can be used in their manufacturing to improve their technological and health-promoting properties.

## 2. Materials and Methods

The present narrative review was conducted by a literature search consulting the PubMed, Web of Science and Scopus databases. The search was limited to English written articles published during the last 18 years, from January 2000 to July 2018. Search terms for general and specific food processing byproducts (“food byproduct”, “food waste”, “food loss”, “vegetable byproduct”, “fruit byproduct”, “grape pomace”, “orange pomace”, “coffee byproduct”, “cheese whey”, “fish byproduct”, “meat byproduct”), were combined with search terms for dairy matrices (“dairy”, “yogurt”, “fermented milk”, “milk”, “cheese”, “butter”, “ice-cream”). In addition, references of relevant reviews and original research articles were manually searched to find out more potential eligible studies. Data on legislation were consulted from the Codex Alimentarius guidelines, the United Nations Food and Agriculture Organization (FAO), the European Food Safety Authority (EFSA) and the Food and Drug Administration (FDA). Data from the FAO Food Balance Sheets regarding worldwide production and losses of the different food commodity groups for the most recent year available (2013) were accessed to study the latest state of global food loss generation. 

The selection of the papers to be included in the review was performed after a thorough study of their content by the authors. The information extracted from the identified references included first author’s name, author affiliation, publication year, dairy product developed, food byproduct used as an ingredient, purpose of adding the food byproduct as an ingredient (technological or health-promoting function) and outcomes. The selection process resulted in the identification of 50 eligible studies which directly addressed the application of a food byproduct as an ingredient in a dairy matrix.

## 3. Byproducts Used as Novel Ingredients in Dairy Foods

Food loss was redefined by FAO in 2014 as “the decrease in quantity and quality of food”. Food waste is considered as part of food loss and refers to discarding or alternative non-food use of food that is safe and nutritious for human consumption along the food chain [7]. Food losses and waste represent an imbalance in the availability and accessibility dimensions in the global food system. Different multifaceted strategies have been proposed by the FAO Committee on Global Food Security to promote the development of a sustainable food system, including food byproduct valorization. In this sense, a reduction in food losses and waste could potentially lead to positive economic, social and environmental outcomes, improving food availability and accessibility, and enhancing a sustainable use of natural resources on which the future production of food depends [8].

The most recent Food Balance Sheets [9] indicate that fruits and vegetables presented the highest values of food losses along the food chain compared to the rest of the commodity groups: cereals, roots and tubers, oilseeds and pulses, meat, fish and seafood, and dairy products. Correspondingly, there has been increasing interest in using fruit and vegetable byproducts as novel ingredients in the development of foods, including dairy products. This focus may be explained by several factors: their impact on the environment, their potential health-promoting phytochemical content, and the fact that plant-derived byproducts and losses mostly occur before household consumption, which makes them still available for reutilization.

**Figure 1:**
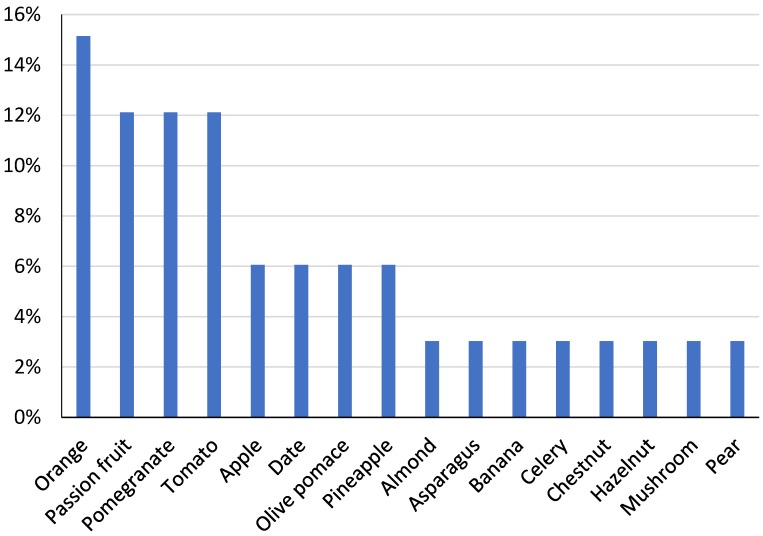
Percentage of research studies that used byproducts from various sources among the fruit and vegetable commodity groups in dairy food manufacturing from 2000 until July 2018 (*n* = 50 studies reviewed).

**Figure 2:**
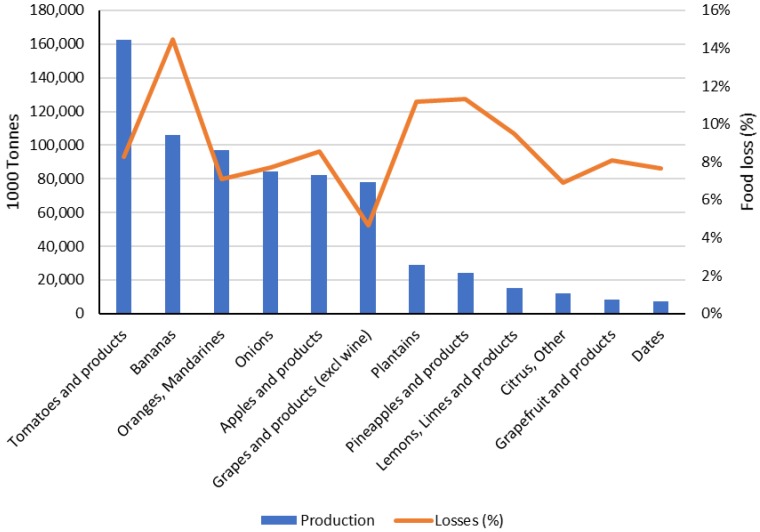
Worldwide food production (1000 tonnes) and its corresponding food loss (%) generated during storage and transportation within the fruit and vegetable commodity groups in 2013. Data obtained from the latest Food Balance Sheets, accessed in 2018 [9].

Of the studies on the development of innovative and health-promoting dairy products using sustainable ingredients published from 2000 to 2018 (*n* = 50 eligible studies), 88% used side-streams from plant materials. Most studies used byproducts from fruits (43%), followed by the application of winery (19%) and vegetable (13%) byproducts. Among fruit and vegetable byproducts, most research has been carried out using citrus and tomato side-streams as ingredients in dairy formulations (Figure 1), which means that efforts have been made to valorize byproducts from food groups that present some of the largest food losses [9]. In 2013 alone, 13.4 and 6.9 million oranges and tomatoes were lost during storage and transportation [9]. It is evident that the amount of food loss is correlated to the amount of the food item produced, but the ratio of food loss within a production chain for a specific item can also help identify which foods are more susceptible to being lost. As seen in Figure 2, bananas, plantains and pineapples have some of the highest loss rates among fruits and vegetables during storage and transportation. This way, further strategies for food loss and waste reduction could focus on using byproducts from these foods as novel ingredients.

Byproducts from meat, fish and seafood contain high amounts of protein, which may be less interesting in dairy food manufacturing as they already contain this compound in their matrix. However, when protein has been needed, it has mostly been obtained from cheese whey, which is a saccharide and protein rich waste generated during cheese production [10]. Using a byproduct from the same industry as a food ingredient not only enhances the sustainability within the dairy industry, but also may translate into fewer sensory difficulties when developing the product due to the similarities of the food matrices.

## 4. Approaches in the Application of Food Byproducts in the Dairy Industry

The exploitation of byproducts generated during food processing or discarded produce as a source of functional compounds and their application in other foods is very much desirable as part of a waste management system [11]. In this review, we divide the applications of food byproducts in dairy foods in two categories: those with technical purposes, which include the improvement of shelf life, safety, stability, sensory quality, etc.; and those with biological purposes, which aim to enhance health-promoting effects for their conversion into functional foods. A summary of the applications so far proposed is shown in Table 1.

### 4.1. Technological Applications of Food Byproducts in Dairy Formulations

One of the major emerging technologies is the application of food byproducts as natural additives. The implementation of this approach could serve a double purpose. As a waste reduction measure, it would enhance sustainability and increase industrial profitability. In addition, it would be possible to fulfill the requirements of consumers concerned about chemical residues in their foods that look for clean-label and naturally-preserved healthy foods [60]. 

The addition of food additives is regulated under Codex Alimentarius guidelines. Therefore, food byproducts used as natural additives must consider current regulations and undertake proper authorization if necessary. In this section, we summarize the ongoing research carried out to apply food byproducts as additives in dairy products.

#### 4.1.1. Use of Byproducts as Antioxidants

The Codex General Standard for Food Additives defines antioxidants as food additives which prolong the shelf-life of foods by protecting against deterioration caused by oxidation [61]. In dairy products, lipid oxidation produces fatty acid hydroperoxides, an intermediary tasteless and odorless compound which can further react with fatty acids leading to the formation of secondary lipid oxidation products and protein damage [62]. These reactions result in the production of off-flavors in milk and dairy products, which are described as cardboardy and metallic [63]. These off-flavors can be detected in raw or pasteurized milk, in any dairy product that has not been flavored, and especially in high-fat products such as butter or ice-cream. Therefore, changes in the properties and palatability of these products can lead to a decrease of consumer acceptability and confidence in dairy products [12].

The susceptibility of milk lipids to oxidation depends on several factors: intrinsic factors, extrinsic factors and their interrelation [62]. Intrinsic factors include the composition of the milk system, which is constituted by a complex mixture of pro-oxidants (transition metals) and antioxidants (tocopherols, uric acid, ascorbic acid), whose relative concentration in milk are related to seasonal, physiological and nutritional effects on the cow [64]. Extrinsic factors that affect lipid oxidation refer to environmental and physical factors (light exposure, temperature, pH, water activity, etc.), and to changes that occur during processing and storage (homogenization, heat treatment, fermentation, proteolysis) [65].

This way, the addition of antioxidants in milk is one of the main methods used for preventing and retarding lipid oxidation. The most commonly applied antioxidants in dairy foods, when their use is not explicitly excluded by legislation, are ascorbates and tocopherols [66].

As an alternative to conventional antioxidants, different bioactive compounds recovered from food byproducts have been used to prevent lipid oxidation of dairy foods and increase their shelf life. These efforts have been made especially in high-fat content dairy foods, such as cheese and butter, but also in yogurts and other dairy products such as milk drinks fortified in omega-3 fatty acids, which have a higher risk of lipid deterioration. *Agaricus blazei* mushroom residue has been added to milk fortified in omega-3 fatty acids, which decreased lipid oxidation when subjected to photooxidation [12]. Wine grape pomace also proved to delay lipid oxidation in yogurt [29], whereas grape seed and pomegranate peel extracts have been applied effectively to protect against lipid oxidation in cheese during storage [56].

Butter contains the largest amount of fat among dairy foods (approximately 80%) and can be kept well for at least 20 days if correctly stored at 10 °C, protecting it from moisture evaporation and light induced photooxidation. However, during cold storage, autoxidation is the main cause of deterioration, which depends on the copper present in the product [67]. Antioxidants from tomato processing byproducts were used as agents against lipid peroxidation in conventional and traditional Tunisian butter, showing a protective action during 4 months of cold storage [11,52]. A protective effect against lipid oxidation during 3 months of cold storage was also shown by adding almond peel extract in whey butter, which contains a higher concentration of unsaturated fatty acids that are more vulnerable to oxidative breakdown [51]. The addition of almond peel extract allowed whey butter storage up to 3 months which showed no significant differences in acceptability scores against milk butter [51].

It is relevant to consider the dosage of the extract added to the food product, as it can affect both the antioxidant behavior of the extract and the final sensory acceptability of the food. The effectiveness of tomato processing byproducts as antioxidants in butter was found to be dose dependent: lower amounts of the extract (400 mg tomato processing byproduct/kg butter) considerably inhibited the formation of oxidation products, extending the shelf life of the product up to two months; whereas greater concentrations of tomato processing byproducts (800 mg tomato processing byproduct/kg butter) showed pro-oxidant properties with detrimental effects on the stability of the butter [52]. This change in the antioxidant/pro-oxidant capacity of certain compounds was also described in other studies [68], which observed that extracts with high concentrations of b-carotene lost their antioxidant effect becoming pro-oxidant, possibly due to long-chain-oxidized products of the carotenoid.

The addition of byproduct extracts can lead to either positive or negative effects on the sensory properties of the final product, depending on the dosage, the type of recovered byproduct compounds and the food matrix in which it is incorporated. In ice-cream, the addition of tomato peel carotenoid concentrations of 4% or higher lowered acceptance scores for flavor, texture, melting quality and color [49]. Tomato byproducts used in a different matrix, butter, significantly improved the product’s appearance after 4 months of cold storage compared to the control butter [11]. Different byproduct extracts, such as grape and pomegranate seed extracts, decreased fat deterioration in sheep yogurt, but their sensory profile was significantly less acceptable than the control samples immediately after yogurt manufacture and after 14 days of storage [39].

#### 4.1.2. Use of Byproducts as Antimicrobials

Preservatives are food additives that prolong food shelf life by protecting against deterioration caused by microorganisms. Different types of preservatives include: antimicrobial antimould antirope and antimycotic agents, antimicrobial synergists, bacteriophage control agents and fungistatic agents [61]. Food byproducts have been used as preservative agents with antimicrobial activity to ensure that manufactured dairy foods remain unspoiled and safe during their whole shelf-life. 

Several studies have shown that food byproducts can be used against spoilage and pathogenic bacteria without interfering with the viability of starter cultures and other microorganisms involved in fermentation processes, ensuring that the quality of the developed products is maintained. The bacterial concentrations required in yogurts and fermented milks by the Codex Alimentarius (10^7^ CFU/g) were still met when different byproducts were added into the food matrix (grape pomace flour and extracts, grape skin and seeds, hazelnut skins, pineapple peels, pomegranate seeds, passion peels, etc.) [22,24,27,28,38,39,45]. In cheese, there is less information on the effect of byproduct addition on molds, yeasts and bacteria during ripening, even though they are essential for the correct development of cheese flavor and texture [69,70]. Winemaking byproducts have been added in Toma-like and cheddar cheese products, showing that their addition did not interfere with starter and nonstarter bacteria nor with cheese proteolysis [59]. Therefore, there is an opportunity to study whether the addition of recovered compounds interferes during ripening in other cheese types that involve the growth of different molds in the cheese rind (soft cheese, natural rind cheese, etc.).

#### 4.1.3. Action Against Dairy Food Spoilage Microorganisms

Milk spoilage is primarily due to the growth of psychrophilic microorganisms that trigger lipolysis and proteolysis reactions of milk fatty acids and proteins, respectively [71]. Lipolysis of milk lipids to free fatty acids and partial glycerides contributes to the desirable flavor of milk and other dairy products, but when present in high concentrations, it can lead to the development of off-flavors. These are described as rancid, butyric, bitter, unclean, soapy and astringent [72]. Once lipolysis produces detectable off-flavors it is not possible to remove them from the product [73]. In addition, the hydrolysis of milk proteins produced by proteases from *Pseudomonads, Aeromonads*, *Serratia* and *Bacillus* spp. also result in the release of off-flavors due to the production of bitter peptides and milk gelation and coagulation [74,75,76].

Milk spoilage is mediated by lipases that are naturally present in milk (lipoprotein lipase) or by lipases and proteases from psychrophilic bacterial contamination occurring during milking, storage and transportation that result in the destabilization of milk during cold storage [62,77]. One of the most important properties of these bacterial enzymes is their heat stability. This is because most of them can retain at least some of their activity after pasteurization or ultra-high temperature (UHT) treatment, even though bacteria are destroyed [63,78]. Therefore, it is important to develop good practices and strategies to minimize the risk, such as achieving a low microbial count in milk before pasteurization as the action of the residual enzymes during storage will shorten the milk’s shelf life [74].

Quality issues and defects associated with excessive lipolysis in dairy products include rancid flavors and poor foaming capacity in pasteurized milk, rancid flavor due to increasing free fatty acids in UHT milk, and spoilage of milk powder during storage. Flavor defects in cheese and butter can be caused by lipolysis before or after manufacture, whereas yogurt is less susceptible to lipolysis defects due to a combination of factors such as low pH, low storage temperature and short shelf life [73]. 

Although different applications of recovered food byproducts are being studied to valorize them as novel food ingredients, there is a lack of information on the effect of the addition of these extracts in the lipolysis or proteolysis of dairy foods. This should be considered, as some additives, such as pepper, promote lipase activity in cheese, producing soapy and rancid off-flavors [73]. To our knowledge, there is only one study that described the effect of byproducts on the hydrolysis of lipids in dairy foods. Tomato processing byproducts were used in butter and ice-cream to prevent lipolysis during 4 months in refrigerated storage [11]. A significant decrease in the liberation of free fatty acids was observed in lycopene added butter after 3 months compared to control butter, suggesting that this extract may exert a protective action against lipolysis.

#### 4.1.4. Action Against Foodborne Pathogens in Dairy Foods

The milk matrix is an ideal media for microorganism proliferation. This also includes pathogenic bacteria, where mycobacteria, *Brucella sp*., *Listeria monocytogenes*, *Staphylococcus aureus* and enterobacteria (including toxigenic *Escherichia coli* and *Salmonella*) are the most frequently found pathogenic bacteria in dairy foods [76]. The origin of pathogen proliferation can be either endogenous (from udder infection) or exogenous (contact with contaminated environment) [79]. Therefore, implementation of Hazard Analysis and Critical Control Points (HACCP) and quality assurance programs through European Union (EU) directives (2004/41/EC, EU 605/2010) on milk hygiene and public health conditions have been put into practice to ensure food safety [80]. 

Milk heat treatment, such as pasteurization or UHT processes, kill pathogenic bacteria. However, inadequate pasteurization or post-pasteurization contamination can cause milk re-contamination if sanitation measures in the processing plant are not sufficient, leading to food poisoning incidences [74]. Outbreaks of food-borne illnesses have been mainly linked to the consumption of raw milk or products made of unpasteurized milk such as raw milk cheeses, whose consumption is continuously growing [81]. Besides not using heat treatment, traditional raw milk cheese producers may not use starter cultures in their elaboration process, which increases the risk of pathogen multiplication as the competitive activity of the lactic acid starter is eliminated [82]. In this sense, the addition of preservatives to dairy products is principally used in cheese. Preservatives may be added during cheese production and ripening to all the edible part of the cheese or only for rind treatment [66,83]. 

The number of dairy food infection outbreaks due to pathogen contamination of other dairy foods is less common, although some cases have been reported for yogurt and fermented milks [84]. In these products the acidity of the matrix acts as a barrier to bacterial growth. However, milk must be pasteurized as some pathogens, such as *E. coli* 0157:H7, can be tolerant to the acid environment [85].

Many studies have analyzed the antimicrobial and antimycotic *in vitro* properties of extracts recovered from food byproducts. The antimicrobial action against foodborne pathogens has been associated with the polyphenols of plant based byproducts, which may penetrate the cell wall causing membrane disruption, damage of membrane proteins and enzymes, and structural changes that lead to bacterial death [86,87,88].

The number of studies analyzing the efficacy of byproduct polyphenolic extracts included in the dairy food matrix on food pathogen control is still limited. Pomegranate peel and grape seed extracts proved to be effective natural preservatives against *Listeria monocytogenes, Staphylococcus aureus* and *Salmonella enterica* in cheese [56]. Pathogen counts in cheese significantly decreased with the byproduct extract treatments. However, the cheese matrix required higher concentrations of the byproduct extracts to efficiently deliver the antibacterial effect compared to the *in vitro* analyses performed in the culture medium. This could be explained by the effect of the micro-architecture of the food matrix. Microbial growth occurs in the aqueous phase of food and is affected by food structure which can restrict the mobility of bacteria. In cheese, which is a gelled emulsion, fat and protein content together with low water content may act as a protective barrier between the bacteria and the extracts, requiring higher concentrations of preservatives to control the growth of pathogens [89].

The addition of herbs and spices in cheese has been part of the cheese culture in many countries for centuries. Some examples include the French Banon covered in chestnut leaves, or the Spanish Majorero cheese with sweet pepper. In this sense, the antimicrobial effect of herbs and spices and their application as cheese preservatives has been more commonly studied [90,91]. This tradition could be used as a cultural advantage for the application of plant-based byproducts as preservative and flavoring agents in innovative cheese developments. As consumers already feel familiar with this type of cheese products, it could increase product acceptability and facilitate its introduction into the market. 

#### 4.1.5. Use of Byproducts as Colorants

Colorants are food additives that add or restore color in foods [61]. Their role is involved in the improvement of the appearance and color of foods, and in the maintenance of their natural color during processing and storage [92]. Color stands as one of the most important quality attributes for the food industry, as it directly affects consumers’ acceptance and food selection [93].

Current market trends include the substitution of synthetic colorants for natural compounds, which has been motivated by consumers’ concern about the safety of synthetic food dyes (side effects, toxicity and allergic reactions), and by the possible health-promoting benefits of natural pigments [94].

Fruit and vegetable byproducts have become an important source of natural pigments as they are colored by green chlorophylls, yellow-orange-red carotenoids, red-blue-purple anthocyanins and red betanins [95]. 

Anthocyanins have been widely extracted from various plant based foods and byproducts, such as radishes, red potatoes, red cabbage, black carrots, purple sweet potatoes, coffee husks, berries, winery byproducts, etc. [96,97,98]. However, their use as food colorants has been limited. The list of anthocyanin colorants in the Codex Alimentarius includes only grape skin extract (E163), and in the FDA, “grape color extract” and “grape skin extract” (enocyanin) [61,99]. 

Anthocyanin application in dairy foods comes with a range of unique coloring challenges, as their stability is affected by changes in pH, fat content in the dairy matrix and manufacturing and storage conditions including extreme temperature and light exposure [97]. Moreover, their use may add specific flavors associated with phenolic compounds. This is the case in some studies where the addition of wine byproducts in yogurt and fermented milks for polyphenol enrichment and color improvement resulted in a decrease in overall liking due to a predominant astringent sensation [29,44,45]. This problem is solved by adding sucrose or other ingredients to the basal recipe to eliminate the astringency. Higher sensory scores in flavor and overall acceptability were reported in wine pomace-fortified fermented milks compared to control samples [30]. The greater acceptability of the polyphenol-fortified samples was probably due to the influence of the intensified color on the perception of taste. Other satisfactory applications of food byproducts as colorants have been reported using anthocyanins from grapes and beetroot betalains. The coloring compounds proved to be stable in semisolid petit-suisse-like cheese probably due to its low water content, slightly acid pH and the low temperature and light-impermeable packaging during storage [100]. 

Carotenoids stand as the major group of compounds used as coloring agents. Their use is widely extended, and the number of authorized carotenoids used as colorants varies depending on each country. Most commercial carotenoids are produced synthetically (β-carotene, astaxanthin, canthaxanthin and zeaxanthin), although some are obtained from natural sources (annatto, paprika, saffron, marigold, tomato, algae) and microbial fermentation [95]. Extraction of lycopene from tomato processing byproducts has been optimized and registered as the food color “E160d” in Europe [61]. In dairy foods, lycopene from tomato byproducts has been applied in the coloring of butter and ice-cream showing a stable reddish color for up to 4 months [11,49].

#### 4.1.6. Use of Byproducts as Texturizing Agents

Texturizing agents are used to add or modify the overall texture and mouth feel of food products by providing creaminess, thickness, viscosity or a stable structure. This category comprises a wide range of food additives including emulsifiers, stabilizers, thickeners and bulking agents [61]. Texturizing agents are commonly used in dairy products. Hydrocolloids are used for stabilizing and thickening purposes in fermented milks, milk drinks, dairy desserts, cream and ice-cream. Phosphates and coagulation agents are also permitted as stabilizers and to aid in the curdling of milk in cheese production, respectively [66,101].

Most hydrocolloids used in dairy foods come from natural origin as they are manufactured by isolation from seaweeds and plant cells [102]. Moreover, many of these hydrocolloids are extracted from plant food wastes, such as pectin, which is commonly isolated from apple pomace and citrus peels, as well as from other fruit and vegetable byproducts such as passion fruit peels, rapeseed cake, olive pomace, grape pomace, onion hulls, etc. [103,104,105,106]. Their application in dairy foods as isolated ingredients is increasing which is a step forward in valorizing underused fractions. However, the isolation of specific compounds generates once again other byproducts. To improve economic and environmental sustainability within the food chain, newer approaches trying to use byproducts as whole ingredients without further processing should be developed. This represents a harder challenge as byproducts used as ingredients comprise a much more complicated matrix than an isolated compound, which could lead to problems associated with product stability and unwanted interaction with other compounds.

In this sense, fewer studies have reported the use of food byproducts as whole ingredients with texturizing purposes. Some examples include the use of liquid fluid whey instead of the generally used powdered form, which showed promising results on the physical quality of white cheese powder [55]. Dietary fiber from orange byproducts was used to maintain the texture of lemon ice-cream when reducing its fat content by 50% [47], and as fat replacers in low-fat yogurt [34]. The authors showed that reducing particle size of the orange dietary fibers by micronization increased their water and oil holding capacities, which are also important functional properties in relation to the facilitation of digestion and absorption of nutrients in the body. 

Texture, rheological parameters and the microstructure of yogurt gels have been analyzed when adding different fibers. A gel structure with large pores and reduced cross-linking between casein micelles in yogurts was observed with 1% of pineapple peel powders, which was associated with lower yogurt firmness and weak rheological properties due to the incompatibility between milk proteins and polysaccharides from the pineapple peel powders [21]. Although the presence of fiber particles always alters yogurt structure, high amounts of passion fruit peel powders or orange byproduct fibers counterbalanced this negative effect and strengthened the casein network possibly due to the water absorption capacity of the fibers [23,35]. This effect of fiber dose and fiber type was also observed in the firmness and spreadability parameters of butter fortified with fibers (from 3% to 5%) from vegetal and fruit wastes: stone pear, celery roots and leaves, spinach and orange albedo [57]. 

### 4.2. Health-Promoting Applications of Food Byproducts in Dairy Formulations

Advances in nutrition and medical science have shown that both nutrients and non-nutrient components of foods are important for maintaining good health. This, together with the increasing knowledge of the biochemical structure and functions of bioactive compounds and their effects on the human body, have led to the rise in popularity of functional foods [1]. Although there is no universally accepted definition of functional foods, they can be described as foods that claim to have health benefits beyond basic nutrition [107]. Functional foods are an increasing market segment aimed at consumers who are taking greater responsibility for their own health and well-being [108]. Simultaneously, diet-related illnesses, such as cardiometabolic diseases including coronary heart disease, stroke, type 2 diabetes and obesity stand as one of the greatest global health and economic burdens of our times, accounting for 31% of all deaths worldwide [109,110]. As part of a healthy dietary pattern and lifestyle, functional foods stand as a promising strategy in non-communicable disease prevention.

Within a scope of food waste reduction, much progress has been made using food byproducts as sources of bioactive compounds or as functional ingredients by themselves for the development of dairy functional products. It must be noted that new food ingredients developed from food byproducts that have not been used for human consumption within the EU prior to 1997 must be subjected to official review and approval according to the European Regulation on Novel Foods and Novel Food Ingredients (258/97). This section summarizes the research that has been carried out using byproducts in the manufacture of health-promoting dairy foods.

#### 4.2.1. Use of Byproducts in the Development of Functional Dairy Foods Containing Polyphenols

Polyphenols are secondary metabolites that are synthesized during normal plant development and in response to stress conditions [111]. Plant phenolics include phenolic acid and its derivatives, flavonoids, lignans and stilbenes [112]. Although phenolic compounds are not considered nutrients, several biological and pharmacological activities have been attributed to dietary polyphenols, including antioxidant, anti-allergic, anti-inflammatory, anti-viral, anti-microbial and anti-carcinogenic effects [113]. These properties play a relevant role in the prevention of several major chronic diseases associated with oxidative stress, such as cardiovascular diseases, cancers, type II diabetes, neurodegenerative diseases and osteoporosis [114]. In this sense, the health-protecting capacity of plant phenolics has become of great interest for researchers, the food industry and consumers. 

Peels, husks, hulls, pods and bran are major processing byproducts of the fruit, vegetable and cereal industry that are considered sources of polyphenols. They have mostly been applied for polyphenol fortification in yogurt and fermented milks. Namely, winemaking byproducts have been used as the main source of polyphenols, including different flours and extracts from grape pomace and other selective fractions, such as grape skins and seeds. This could be justified both by the fact that black grapes stand among the richest dietary sources of polyphenols [115,116] and by the high amount of grape losses generated during processing and conversion into wine, storage and transportation, which reached 3.6 million in 2013 [9]. Other byproducts from fruits, nuts, vegetables and cereals have also been used as sources of polyphenols for the development of fermented milks and yogurts. These byproducts included pomegranate seeds and peels, almond peels, hazelnut skins, olive pomace and rice bran [12,14,18,37,38,39]. 

The addition of polyphenols to dairy foods other than yogurt and fermented milks has received less attention. Wine pomace byproducts have also been the major source of polyphenols used to formulate cheese [53,56,58,59] and ice-cream [50], although other phenol byproduct sources have recently been studied in spreadable cheese (tomato peels, broccoli stems and leaves, corn bran and artichoke external leaves) [53]. The application of broccoli stems in spreadable cheese is particularly interesting, as it could increase glucosinolate content in the product, which are compounds also associated with beneficial health properties [117].

Wine pomace flours have been directly used as ingredients in fermented milk and yogurt development [28,29,45]. The advantage of using powders instead of extracts from the byproducts is that less processing is required, which is a more sustainable approach as it consumes less energy and does not generate secondary byproducts. On the other hand, the disadvantage of using powders is that higher doses are needed to achieve significant polyphenol fortification levels, which penalizes the organoleptic properties of the products. That is why many studies have switched towards using extracts from wine pomace [27,30,39,43].

In this sense, product formulations with a compromise between functional properties and sensory acceptance need to be developed. In foods, polyphenols may contribute to the bitterness, astringency, color, flavor and odor of the products [118]. Polyphenols are associated with the precipitation of salivary glycoproteins and mucopolysaccharides on the tongue, resulting in roughness and dryness on the palate [119]. This is why several studies have reported an inverse relation between polyphenol dosage and consumer acceptance in dairy products [29,38,44,59]. A decrease in the overall acceptance of yogurts with 6% added polyphenols from grape skin flours [45] and of yogurts with 1% and 2% grape pomace powders [120] was observed compared to yogurt formulations with lower doses due to flavor, texture and consistency parameters.

In order to mask the negative sensory effects of polyphenols, several researchers have evaluated their use together with other ingredients. In yogurt and fermented milk fortification with wine pomace byproducts, the best acceptance scores were obtained when polyphenols were added in combination with sucrose (5%), oligofructose (0.5% to 0.667%) or grape juice (0.167% to 0.5% and 15%) [30,44].

Besides the sensory and quality challenges associated with the addition of polyphenols in the dairy matrix, the bioaccessibility and bioavailability of the bioactive compounds should be taken into consideration to truly establish whether the wanted biological health effects are being met. Evidence suggests that polyphenols are absorbed in a relatively low amount. Most polyphenols are poorly absorbed in the gastrointestinal track, reaching the colon where they are metabolized by colonic microbiota. These metabolites are responsible for the biological activities associated with polyphenols [121]. The resulting bioactivity will depend both on the interactions between polyphenols and other macromolecules (lipids, proteins and carbohydrates), which will affect their bioaccessibility and bioavailability, and on the specific microbiota present in each individual’s colon, which can give rise to different phenolic metabolites [117,122]. Therefore, further knowledge on the food matrix and food interaction together with the role of gut microbiota on the metabolism and activation of the dietary constituents, will provide original ideas for the development of new functional foods, in which a combination of plant-derived food ingredients with the appropriate bacterial strains will lead to improved biological activity for a specific food product [117].

#### 4.2.2. Use of Byproducts in the Development of Functional Dairy Foods Containing Dietary Fiber

Plant-derived byproducts, such as seed, skins, pods, peels, pomace, hulls, husks, cores, stores, etc., are known sources of bioactive compounds and nutrients including dietary fiber [1,123], whose caloric value has been estimated at 2 kcal per g (FDA, 2018). The European Food Safety Authority (2010) [124] defines this nutrient as non-digestible carbohydrates, including non-starch polysaccharides, resistant starch and oligosaccharides, and lignin. A terminology often encountered is the classification of dietary fiber as “soluble” or “insoluble” [125]. Therefore, the physicochemical properties of the different dietary fibers can be determinant when selecting their applications.

Worldwide and country-specific governmental institutions confirmed that there is evidence of health benefits associated with consumption of diets rich in fiber-containing foods, and recommendations on the intake of dietary fiber range between 25 g to 38 g per day [126]. Health benefits have been related to a reduced risk of coronary heart disease, intestinal disorders, type 2 diabetes and improved weight maintenance [127,128,129]. 

Product innovations have been focused on increasing the fiber content of dairy foods with two purposes: to help consumers achieve the daily recommended intake of dietary fiber, and as a marketing strategy to add a nutritional claim on the food package. The European Parliament and Council, (2006) (Regulation No. 1924/2006) [130] stated that the nutritional claim “source of fiber” or “high in fiber” can only be made when the product contains at least 3% (or 1.5 g of fiber per 100 kcal) or 6% (or 3 g of fiber per 100 kcal) dietary fiber, respectively. Bearing this in mind, several researchers have used dietary fiber concentrations ranging from 2.5% to 10% to evaluate its feasibility as an ingredient in dairy products, as an increase in concentrations of dietary fiber in foods can lead to changes in the resultant nutritional, textural, rheological, and sensory properties of the developed products [131].

Development of dairy foods fortified with high contents of dietary fiber have mostly been carried out in yogurt and fermented milks. Available studies have used a wide variety of plant-origin sources derived from fruit and vegetable industry byproducts. Water soluble soybean polysaccharides from okara, which is the byproduct of tofu, soymilk and soybean protein isolate, were used in the development of ice-cream, pudding and a milk-based beverage [26]. Optimal sensory acceptance of products was achieved in milk beverages and pudding with 4% dietary fiber, and in ice-cream with 2% dietary fiber. Higher dietary fiber doses were rejected as consumers considered the foods too thick when evaluated using Just About Right (JAR) scales. In fermented milks and yogurts, fiber from apple pomace (3% to 10%), date byproducts (1.5% to 4.5%) and hazelnut skins (3% to 6%) were used in the development of dietary fiber-fortified foods [20,31,38]. In these cases, optimal sensory acceptance of the products was obtained at 3% fiber addition from hazelnut skins and dates, and 5% fiber addition from apple pomace. 

These examples demonstrate that it is possible to increase the doses of dietary fiber for the development of dairy products with optimal sensory acceptance that could be labeled as “source of fiber” on their package. Achieving a “high in fiber” label may be more problematic both from a technological and biological point of view, as textures may be too thick, and consumption of high content dietary fiber products may cause potential secondary effects from carbohydrate fermentation including bloating, distension, flatulence, loose stools and increased stool frequency [132]. 

Other studies have successfully fortified yogurts and fermented milks with dietary fiber from other byproduct sources, such as orange, passion fruit and asparagus byproducts, but in lower doses (0.6%–1%), which also contribute to increasing the daily intake of dietary fiber in consumers’ diets and potentially promote associated health benefits, but do not achieve a nutritional claim [24,25,36,42]. 

Lower doses of dietary fiber from food byproducts have also been used in the development of fermented milks to protect probiotics and enhance their viability. It is well documented that probiotic bacteria grow slowly in milk because they are devoid of proteolytic enzymes [133]. Therefore, milk solids supplementation is a good practice to improve probiotic growth during fermentation and favor their viability in the product [134]. Rice bran, olive and wine pomace, cheese whey, pineapple, apple, banana and chestnut byproducts have been used for probiotic protection in fermented milks [13,14,17,18,19,22,27,28,30]. To our knowledge, the only attempt to use byproducts to promote probiotic viability in a different dairy matrix has been using wheat bran in cheese [54]. 

In addition to enhancing probiotic viability, probiotic strains can act synergistically with specific types of fiber during fermentation to improve the fatty acid composition in fermented milks. This is because some strains of bacteria are able to change the fatty acid profile of milk during fermentation and produce functional fatty acids, including conjugated fatty acids, as the result of their growth and metabolism [135]. Moreover, the addition of other ingredients into the milk, such as prebiotics, can further increase the content of functional fatty acids in fermented milks [136]. In a study using *Lactobacillus acidophilus* and *Bifidobacterium animals* subsp. *lactis* strains, the addition of banana fiber significantly increased α-linoleic acid content, whereas passion fruit fiber promoted the increase of conjugated linoleic acids in probiotic yogurts [19]. Therefore, further studies should focus on the probiotic-fiber synergistic effect to improve the nutritional quality of dairy products, as the application of dietary fiber from fruit byproducts could be a more cost-effective and sustainable option than the addition of conjugated linoleic acids precursors and commercial soluble fiber that are normally used to improve the fatty acid profile of yogurts.

#### 4.2.3. Use of Animal Origin Byproducts in the Development of Functional Dairy Foods

Ingredients derived from animal origin byproducts have been used in the development of dairy foods fortified in omega-3 fatty acids and dairy foods with a high protein content. Fish oil extracted from fish wastes is an excellent source of many unsaturated fatty acids, including long chain omega-3 cis-5,8,11,15,17-eicosapentaenoic acid (EPA) and cis-4,7,10,13,16,19-docosahexaenoic acid (DHA) [137]. However, its application in food formulations fortified in omega-3 is limited because of its easy oxidation and strong odor [41]. Successful attempts to develop yogurts containing omega-3 that had sensory attributes similar to plain yogurt were obtained by encapsulating fish oil in nano-liposomes [40], and adding a fish oil/γ-oryzanol nanoemulsion to yogurt [41].

Functional dairy foods using whey proteins obtained from cheese processing have been widely used as fat replacers and in the development of dairy foods with the nutritional claim “source of protein” or “high in protein” that are already commercially available. Problems associated with using whey proteins and sodium caseinate as fat replacers in yogurt included powdery taste, excessive acid development from lactose fermentation, higher syneresis, excessive firmness and grainy texture [33]. Improved texture parameters have been achieved in low fat yogurts and low fat probiotic yogurts with added whey-buttermilk protein aggregates, whey protein concentrate and heat-treated whey protein concentrates [15,16,17]. 

## 5. Sensory Challenges and Consumer Perspective of Using Byproducts in Dairy Foods

Towards the end of the nineties, consumer acceptance was both referred to as the key success factor for functional foods and the top priority for further research [138]. Since then, several authors have tried to cover this research gap, focusing on sensory and consumer science of functional foods. The latest findings have shown that the perceived importance of food for health is still increasing, but that consumers’ critical attitude towards functional foods is also increasing, which translates into lower willingness to compromise on taste for health [139]. 

The current approach to the development of functional foods using byproducts as novel ingredients has focused on selecting specific concentrations of the byproducts to improve the technological and health-promoting properties of the products, and afterwards, evaluating their sensory acceptance. Not all studies included sensory or consumer analyses of the developed product, and when done, many studies were short on the number of volunteers to achieve significant conclusions. This context reflects that the gap in sensory and consumer research is still present, and that further analyses in this field need to be included in the academic and industry sectors to respond to the good-tasting functional food demand. 

The challenge of developing good-tasting functional foods within the dairy industry increases when using food byproducts. Several authors have reported organoleptic issues associated with the use of byproducts in dairy foods, mainly due to the acrid, astringent, bitter or salty off-flavors inherent to plant-based phytonutrients [29,45,140]. In addition, there is a lack of information on the consumer’s perspective of using food byproducts as ingredients in other foods. The possible concerns regarding food quality and safety that may arise, as well as the importance of sustainability as a driver in food choice should be investigated. 

Bearing in mind that the functional food segment is a highly competitive and continuously changing market, using food byproducts as ingredients could be regarded as an opportunity for product differentiation. Further research should focus on the development of innovative flavors and textures to achieve more palatable foods, as well as on suitable marketing strategies to place these healthy and sustainable products in the market. 

## 6. Conclusions

The applications described in this review show the high potential of valorizing food byproducts for the development of innovative and healthy dairy foods. Byproducts used as sustainable ingredients or sources of bioactive compounds have been shown to be effective for a wide range of technological and nutritional purposes in dairy product manufacture. This approach not only takes a step forward to waste reduction in the food chain, but also offers new ways to diversify the production of dairy foods, creating the possibility of satisfying a market niche based on functional and sustainable products. It is crucial that food technologists, nutritionists and sensory scientists work together to face the challenge of developing more palatable and well accepted foods. Moreover, it is necessary to analyze the consumer’s perception and potential food safety concerns on the use of byproducts in food formulations, and specifically, for the dairy food segment.

## Figures and Tables

**Table 1:** Dairy foods found in the literature (from 2000 to July 2018, *n* = 49) developed using food processing byproducts as sustainable ingredients.

Dairy Product	Food Industry	Byproduct	Doses	Function	Reference
Dairy beverage	Vegetable	Mushroom residue	1, 2 and 3 g/kg	Technological (antioxidant) Health-promoting (source of phenols)	Vital et al., 2017 [12]
		Olive vegetable water	100 mg/L to 200 mg/L	Health-promoting (source of phenols, probiotic protection)	Servili et al., 2011 [13]
Fermented milk	Cereal	Rice bran	1% to 3%	Health-promoting (source of fiber and phenols, probiotic protection)	Demirci et al., 2017 [14]
	Dairy	Whey protein	2%	Technological (texturizing agent) Health-promoting (source of protein)	Akalin et al., 2012 [15]
		Whey protein and buttermilk	0% to 100% replacement of skim milk powder	Technological (texturizing agent) Health-promoting (source of protein)	Saffon et al., 2013 [16]
		Whey protein	8% to 14%	Technological (texturizing agent) Health-promoting (source of protein, probiotic protection)	Zhang et al., 2015 [17]
	Fruit	Chestnut flour	2%	Health-promoting (source of phenols, probiotic protection)	Ozcan et al., 2016 [18]
		Apple	1%	Health-promoting (source of fiber, probiotic protection)	Do Espírito Santo et al., 2012 [19]
		Apple pomace	2.5% to 10%	Health-promoting (source of fiber)	Issar et al., 2016 [20]
		Banana	1%	Health-promoting (source of fiber, probiotic protection)	Do Espírito Santo et al., 2012 [19]
		Pineapple peel powder	1%	Technological (texturizing agent)	Sah et al., 2016 [21]
		Pineapple peel powder	1%	Health-promoting (probiotic protection)	Sah et al., 2015 [22]
		Passion fruit peels	1%	Technological (texturizing agent)	Espírito-Santo et al., 2012 [23]
		Passion fruit peels	0.7%	Health-promoting (source of fiber)	Do Espírito Santo et al., 2012 [24]
	Vegetable	Passion fruit	1%	Health-promoting (source of fiber, probiotic protection)	Do Espírito Santo et al., 2012 [19]
		Passion fruit peels	1%	Health-promoting (source of fiber)	Perina et al., 2015 [25]
		Okara	3% to 10%	Health-promoting (source of fiber)	Chen et al., 2010 [26]
		Olive pomace	100 mg/L TPC	Health-promoting (source of phenols, probiotic protection)	Aliakbarian et al., 2015 [27]
	Winery	Wine pomace extract	100 mg/L TPC	Health-promoting (source of phenols, probiotic protection)	Aliakbarian et al., 2015 [27]
		Grape marc flour	10, 20 and 50 g/L	Health-promoting (source of phenols, probiotic protection)	Aliakbarian et al., 2013 [28]
		Wine pomace extract and flour	1% to 3% 1% to 2%	Technological (antioxidant, colorant) Health-promoting (source of fiber and phenols)	Tseng and Zhao 2013 [29]
		Wine pomace extract	788 mg GAE/100 g	Health-promoting (source of phenols, probiotic protection)	Dos Santos et al., 2017 [30]
		Wine pomace flour	10, 20 and 50 g/L	Health-promoting (source of phenols, probiotic protection)	Frumento et al., 2013 [28]
Yogurt	Cereal	Wheat bran	1.5%	Health-promoting (source of fiber)	Hashim et al., 2009 [31]
		Rice bran	0.2% to 0.6%	Technological (colorant)	Nontasan et al. 2012 [32]
	Dairy	Whey protein	3.3, 5 and 10 g/L	Technological (texturizing agent) Health-promoting (source of protein)	Sandoval-Castilla et al., 2004 [33]
	Fruit	Date byproducts	1.5% to 4.5%	Health-promoting (source of fiber)	Hashim et al., 2009 [31]
		Orange peels, pulp, seed powders	1% to 3%	Technological (texturizing agent)	Yi et al., 2014 [34]
		Orange byproducts	0.2 to 1 g/mL	Technological (texturizing agent)	Sendra et al., 2010 [35]
		Orange albedo, flavedo and pulp powders	0.6% to 1%	Health-promoting (source of fiber)	García-Pérez et al., 2005 [36]
		Pomegranate peel extract	5% to 35%	Health-promoting (source of phenols)	El Said et al., 2014 [37]
		Hazelnut skin powder	3% to 6%	Health-promoting (source of fiber)	Bertolino et al., 2015 [38]
		Pomegranate seed	25 mg/L	Technological (antioxidant)	Ersöz et al., 2011 [39]
	Marine	Fish oil	15 mL/100 g	Health-promoting (source of omega-3)	Ghorbanzade et al., 2017 [40]
		Fish oil	13 g/100 g	Health-promoting (source of omega-3)	Zhong et al., 2018 [41]
	Vegetable	Asparagus byproducts	1%	Health-promoting (source of fiber)	Sanz et al., 2008 [42]
	Winery	Grape seed extract	100 mg/150 g	Health-promoting (source of phenols)	Chouchouli et al., 2013 [43]
		Grape skin flour	0.167 to 1 g/100 g	Health-promoting (source of phenols)	Karnopp et al., 2017 [44]
		Grape skin flour	60 g/kg	Health-promoting (source of phenols)	Marchiani et al., 2016 [45]
		Grape seed	25 mg/L	Technological (antioxidant)	Ersöz et al., 2011 [39]
Dairy dessert	Fruit	Date byproduct	0.5, 1 and 2 ratio dried date powder/date syrup	Technological (texturizing agent)Health-promoting (source of phenols)	Jridi et al., 2015 [46]
	Vegetable	Okara	3% to 10%	Health-promoting (source of fiber)	Chen et al., 2010 [26]
Ice-cream	Fruit	Orange peels, pulp, seed powders	1% to 1.5%	Technological (texturizing agent)	Crizel et al., 2014 [47]
		Pomegranate peels	0.1% and 0.4%	Health-promoting (source of phenols)	Çam et al., 2013 [48]
	Vegetable	Lycopene from tomato byproducts	70 mg/kg	Technological (antioxidant, colorant, antimicrobial)	Kaur et al., 2011 [11]
		Carotenoids from tomato peels	1% to 5%	Technological (antioxidant, colorant)	Rizk et al., 2014 [49]
	Winery	Grape wine lees	50, 100 and 150 g/kg	Health-promoting (source of phenols)	Hwang et al., 2009 [50]
Butter	Fruit	Almond peel extract	100 ppm to 400 ppm	Technological (antioxidant)	Nadeem et al., 2014 [51]
	Vegetable	Lycopene from tomato byproducts	20 mg/kg	Technological (antioxidant, colorant, antimicrobial)	Kaur et al., 2011 [11]
		Tomato processing byproduct	400 and 800 mg/kg	Technological (antioxidant)	Abid et al., 2017 [52]
Cheese	Cereal	Corn bran	5%	Health-promoting (source of phenols)	Lucera et al., 2018 [53]
		Wheat bran	10 g/500g	Health-promoting (probiotic protection)	Terpou et al., 2018 [54]
	Dairy	Fluid whey	Water substitution	Technological (texturizing agent)	Erbay et al., 2015 [55]
	Fruit	Pomegranate peel	100 mL/25 g	Technological (antioxidant, antimicrobial)	Shan et al., 2011 [56]
		Orange byproduct fibers	3% to 5%	Technological (texturizing agent)	Saraç and Dogan 2016 [57]
		Pear stones	3% to 5%	Technological (texturizing agent)	Saraç and Dogan 2016 [57]
	Vegetable	Spinach	3% to 5%	Technological (texturizing agent)	Saraç and Dogan 2016 [57]
		Celery byproduct fibers	3% to 5%	Technological (texturizing agent)	Saraç and Dogan 2016 [57]
		Okara	1% to 4%	Health-promoting (source of fiber)	Chen et al., 2010 [26]
		Tomato peels	5%	Health-promoting (source of phenols)	Lucera et al., 2018 [53]
		Broccoli stems and leaves	5%	Health-promoting (source of phenols)	Lucera et al., 2018 [53]
		Artichoke external leaves	5%	Health-promoting (source of phenols)	Lucera et al., 2018 [53]
	Winery	Grape seed	100 mL/25 g	Technological (antioxidant, antimicrobial)	Shan et al., 2011 [56]
		Wine pomace, skin and seed extracts	0.1, 0.2 and 0.3 wt/vol	Health-promoting (source of phenols)	Da Silva et al., 2015 [58]
		Wine pomace flour	0.8 and 1.6 w/w	Health-promoting (source of phenols)	Marchiani et al., 2015 [59]
		Grape pomace	5%	Health-promoting (source of phenols)	Lucera et al., 2018 [53]
